# In-Depth Analysis of the Paramagnetic Properties in DHI/DHICA-Controlled Eumelanin

**DOI:** 10.1021/acsomega.5c08896

**Published:** 2025-11-07

**Authors:** João V. Paulin, João P. Cachaneski-Lopes, Emanuele Carrella, Alessandro Pezzella, Augusto Batagin-Neto, Carlos F. O. Graeff

**Affiliations:** † School of Sciences, Department of Physics and Meteorology, 28108 São Paulo State University (UNESP), Bauru, São Paulo 17033-360, Brazil; ‡ Graduate Program in Materials Science and Technology (POSMAT), São Paulo State University (UNESP), Bauru, São Paulo 17033-360, Brazil; § Department of Chemical Sciences, 9307 University of Naples Federico II, via Cinthia 4, Naples 80126, Italy; ∥ Department of Physics Ettore Pancini, University of Naples Federico II, Via Vicinale Cupa Cintia, 21, Naples 80126, Italy; ⊥ Institute for Polymers Composites and Biomaterials (IPCB) CNR, Via Campi Flegrei 34, IT, Naples, Pozzuoli 80078, Italy; # National Interuniversity Consortium of Materials Science and Technology (INSTM), Piazza S. Marco, 4, Florence, Naples 50121, Italy; ¶ Institute of Sciences and Engineering, São Paulo State University (UNESP), Itapeva, São Paulo 18409-010, Brazil; ∇ Bioelectronics Task Force at University of Naples Federico II, Naples 80126, Italy

## Abstract

Eumelanin, a naturally occurring pigment derived from the oxidative polymerization of 5,6-dihydroxyindole (DHI) and 5,6-dihydroxyindole-2-carboxylic acid (DHICA), has been extensively studied for its role in human biology and its potential applications in biomedical and sustainable electronic fields. By employing X-band electron paramagnetic resonance spectroscopy, this study focuses on eumelanin with different DHI/DHICA ratios, directly comparing monomeric and polymeric forms. The analysis reveals significant differences in the paramagnetic profiles, with changes in spin dynamics and relaxation times closely linked to the distinct macrostructures formed during polymerization. These findings offer valuable insights into how the molecular architecture of eumelanin influences its paramagnetic environments.

## Graphical abstract

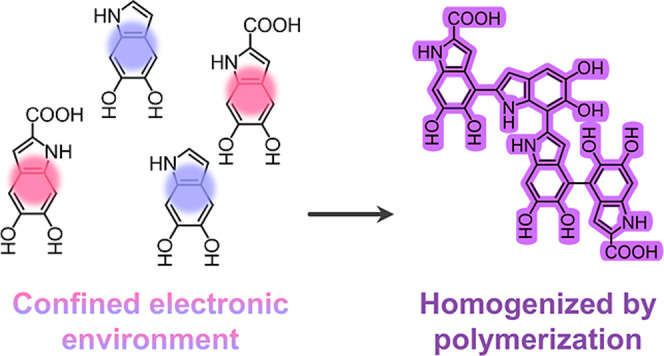

## 1 Introduction

Eumelanin, a prominent natural pigment, has garnered significant attention due to its complex structure and diverse functional properties.^1,2^ It is most known for determining the coloration of human hair, skin, and eyes.^1–3^ However, it can also protect against ultraviolet radiation and scavenge harmful free radicals, safeguarding cellular health.^1–3^ This protective functionality underscores the pigment’s crucial role in preserving the integrity of human tissues exposed to environmental stressors. Additionally, due to eumelanin’s biocompatibility^4–6^ and biodegradability,^7,8^ it has also gained a lot of attention in biomedical and sustainable (bio)­electronics.^1,2^

Structurally, eumelanin is a complex macrostructure derived from the oxidative polymerization of 5,6-dihydroxyindole (DHI) and 5,6-dihydroxyindole-2-carboxylic acid (DHICA) in different redox states, Figure 1a. The intricate arrangement of these monomers at C2, C3, C4, and C7 positions results in a heterogeneous, amorphous network that contributes to eumelanin’s broad absorbance throughout the UV–visible region,^9^ strong nonradiative relaxation of photoexcited electronic states,^10^ metal chelation^1,11^ and hydration-dependent conductivity.^12–16^

**Figure 1:**
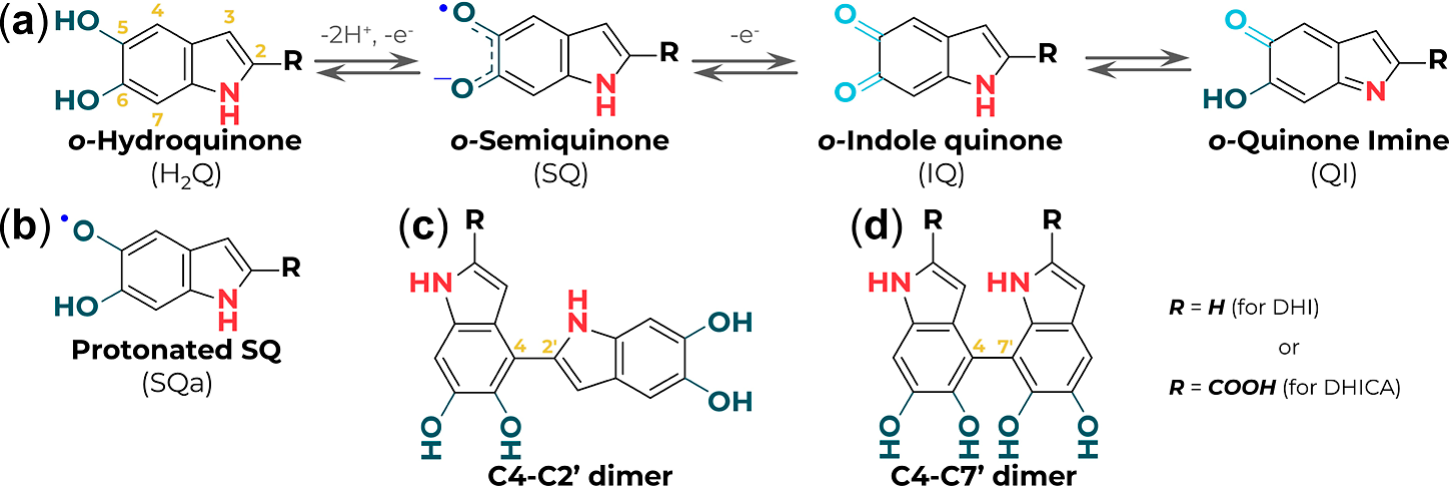
(a) Oxidation and reduction forms of the eumelanin monomeric precursors. (b) Protonated semiquinone (SQa). (c) C4–C2′ and (d) C4–C7′ dimer structures. 5,6-Dihydroxyindole (DHI) is represented by R = H and 5,6-dihydroxyindole-2-carboxylic acid (DHICA) by R = COOH.

A particularly intriguing property of eumelanin is its persistent paramagnetic signal, which is among its most extensively studied properties and can be easily detected using continuous-wave (CW) electron paramagnetic resonance (EPR) under various experimental conditions.^17^ Today, it is understood that eumelanin’s paramagnetic system is composed of two types of free radicals: an intrinsic free radical, originating from the development of its macrostructure, and an extrinsic free radical, typically linked to the redox state of the eumelanin monomers.^17^ The concentration of these extrinsic free radicals is determined by the comproportionation equilibrium reaction (QH_2_ + IQ + 2H_2_O ⇄ 2SQ + 2H_3_O^+^). The intrinsic free radicals are carbon-centered (CCR), with their spin density distributed on carbon atoms. In contrast, the extrinsic free radicals are semiquinone-free radicals (SFR), where the spin density resides on oxygen atoms.^17^ Additionally, these species can be differentiated by their line shapes and *g*-values, approximately 2.0030 for CCR and 2.0050 for SFR.^17^

Although the CCR and SFR labels help classify eumelanin’s paramagnetic species, each category may include a range of radicals with slightly different electronic structures.^18^ This diversity reflects how local factors can modify the spin distribution and lead to variations in the EPR response.

Here, we perform an X-band CW-EPR investigation of chemically-controlled eumelanin with varying ratios of DHI and DHICA. This approach is particularly important because differences in synthetic conditions can lead to variations in the proportion of DHICA incorporated into the final polymer.^1,17^ By systematically controlling the DHI/DHICA ratio, we aim to investigate how compositional differences influence the electronic and magnetic properties of eumelanin.

Additionally, we aim to directly compare the monomeric and polymeric forms, as understanding the relationship between molecular structure and material properties is fundamental in the study of eumelanin. The stable radicals present in eumelanin have been indirectly linked to its electrical and conductive properties.^12,15,18,19^ Therefore, the comparison between monomer and polymer is particularly valuable for revealing how polymerization influences eumelanin’s electronic and paramagnetic characteristics. By examining monomers, we gain insights into the fundamental interactions at the molecular level, while the study of polymers allows us to observe how these interactions evolve as the material becomes more complex and structured.

## 2 Materials and Methods

### 2.1 Sample Preparation

#### 2.1.1 Materials

All commercially available reagents were purchased from Sigma-Aldrich and used as received. All the solvents were of analytical grade.

#### 2.1.2 DHI Synthesis

DHI was prepared following a previously reported two-step method.^20^

Step I: in a deoxygenated solution (purged with nitrogen for at least 30 min) of l-DOPA (3,4-dihydroxy-l-phenylalanine, 2.0 g, 10.0 mmol) in 1.0 L of distilled water, a mixture of potassium bicarbonate (KHCO_3_; 5.0 g) and potassium hexacyanoferrate­(III) (K_3_[Fe­(CN)_6_]; 13.2 g, 40.0 mmol) in distilled water (60.0 mL) was added dropwise, using a pressure-equalizing dropping funnel, under a nitrogen flux.

Step II: after 2–3 h, when the solution’s color changed from red to brown-black, sodium dithionite (300 mg) was added, and the solution was slowly adjusted to pH 4 using 3 M hydrochloric acid (HCl). The resulting mixture was then extracted three times with ethyl acetate (300 mL per extraction), and the organic phases were dried and concentrated using a rotary evaporator. Finally, the residue was washed twice with benzene. A greyish solid with an 80% yield was obtained.

#### 2.1.3 DHICA Synthesis

A similar methodology to that used for DHI synthesis was applied.^20^ Step I was identical to DHI synthesis; however, in step II, immediately after the initial addition of KHCO_3_ and K_3_[Fe­(CN)_6_], 50 mL of 3 M sodium hydroxide (NaOH), previously deoxygenated under nitrogen, was added. After 15 min, when the red color of the solution had completely disappeared, the mixture was sequentially treated with sodium metabisulphite (300 mg), adjusted to pH 2 with 3 M HCl, and extracted with ethyl acetate (3 × 300 mL). The organic phases were then dried over sodium sulfate and concentrated under a flux of nitrogen, obtaining a greyish powder with an 88% yield.

### 2.2 Polymerization and DHI/DHICA Mixtures

Ten mg of a DHI/DHICA mixture in the appropriate ratio (1:0, 7:3, 1:1, 3:7, 0:1% wt) was dissolved in 500 μL of a 1:1% v ethyl acetate (Cinética, 99%) and methanol-dry (Chemco, 99.8%) solution and then drop-cast onto precleaned glass substrates (2.5 cm × 2.5 cm). After drying, the deposited films were exposed to ammonium hydroxide (Neon, 28–30%) vapor for 18 h as part of the ammonia-induced solid-state polymerization (AISSP) process.^6^ The 04 films were dried at 100 °C for 10 min in the air, then scraped. The resulting powders were combined into a single batch to ensure sample homogeneity and reduce possible batch-to-batch variations. The final materials were stored at room temperature.

### 2.3 Continuous-Wave X-Band EPR Measurements

The EPR spectra were recorded in the solid-state using an X-band CW spectrometer, MiniScope MS300 (Magnettech, Berlin, Germany). A Frequency Counter 53181A RF monitored the microwave frequency for each measurement. The CW-EPR spectra were acquired with a modulation amplitude of 80 μT, scan width of 6 mT and microwave power of 0.1 mW using 10 passes. *g*-Values were corrected against the measured value of the 2,2-diphenyl-1-picrylhydrazyl (DPPH) standard marker in *g* = 2.0036. All measurements were performed at room temperature (*T* ≈ 298 K) and without hydration control. It should be noted that, although the water content does not alter the line-shape features, it can affect the intensity of the EPR signal.^21^ Consequently, the lack of hydration control prevents an accurate determination of the spin density in the samples.

In addition, the microwave powers ranged from 0.10 to 31.62 mW, using the same experimental conditions, to analyze power saturation and evaluate spin relaxation behavior.

### 2.4 Data Analysis

To analyze the power saturation experiments, we employed Altenbach’s methodology as described in eq 1.^22,23^

1 $$A=S\frac{\sqrt{P}}{{\left[1+\left(2^{1/\varepsilon }-1\right)\frac{P}{P_{1/2}}\right]}^{\varepsilon }}$$

where *A* represents the signal intensity and *S* is the proportionality constant that describes the initial rate of increase in signal amplitude as a function of the square root of the microwave power (MWP) within the unsaturated linear range. *P*_1/2_ determines the half-power saturation value, and ε represents the degree of saturation homogeneity. ε range between 0.5 (indicating inhomogeneous saturation) and 1.5 (indicating homogeneous saturation). The numerical fitting was done using OriginPro 9.0. *S* and *P*_1/2_ were freely adjusted during the fitting process, while ε was allowed to vary within its defined range.

The rate at which the spins can exchange energy with the lattice can be described by the spin–lattice relaxation time^24–26^ following $T_{1}\propto {\left(P_{1/2}\right)}^{-1}$ . On the other hand, spin–spin relaxation time (*T*_2_) can be directly obtained using the Bloch equation from the CW-EPR line width following eq 2 ^24–26^

2 $$T_{2}=\frac{2}{\sqrt{3}\gamma {\Delta B}_{PP}}=\frac{1.3131\times 10^{-7}}{g{\Delta B}_{PP}}$$

where γ is the electron magnetogyric ratio, Δ*B*_PP_ is the peak-to-peak line width and *g* is the *g*-value. $T_{2}^{-1*}=\frac{1}{T_{2}}$ is referred to as the relaxation rate.

The CW-EPR spectra simulation analysis was carried out using the free EasySpin computational package (v. 6.0.2),^27^ following previously published works.^21,28,29^ The simulations were performed with the aid of a pepper routine. Various line shape broadening (Lorentzian, Gaussian, and Voigtian), anisotropies (isotropic or axial symmetry), and number of species (one, two, or three) were considered, as shown in Figure S1 in the Supporting Information. The optimal simulations used Lorentzian lines and isotropic symmetry.

### 2.5 Electronic Structure Calculations

This study considered radical semiquinone (SQ) as well as charged, anionic and cationic, (HQ, IQ and QI) monomer units. Oligomeric structures (up to 6 units) DHI and DHICA, linked via C4–C2 (DHI^4^/_2_DHI) or C4–C7 (DHI^4^/_7_DHI and DHICA^4^/_7_DHICA) positions, were also evaluated to underline the effect of oligomerization on the EPR response. For all the oligomers, one SQ radical unit was attached to the beginning of the HQ-based chains.

The monomeric and oligomeric structures were designed using the GaussView computational package.^30^ These structures were optimized within the density functional theory (DFT) framework, employing the B3LYP exchange–correlation (XC) functional and the 6-311G­(d,p) basis set for all atoms. The EPR analysis was performed using Orca software,^31^ applying the B3LYP XC functional and the EPR-II basis set^32^ *in vacuo* (to simulate dry samples). The B3LYP/EPR-II approach has been validated in the literature and is known to reproduce experimental *g*-values with good accuracy at moderate computational cost.^33^ Explicit spin–orbit coupling (SOC) beyond the perturbative orbital Zeeman/one-electron (1e) SOC term was not considered,^34^ given the nature of the compounds (composed only of light atoms). Additional single-point calculations were carried out for representative monomer-based radicals at distinct levels of theory (PBE0/EPR-II and B3LYP/EPR-II + 2e-SOC terms) for comparative purposes (see Section S2 in Supporting Information).

Hydrogens were removed from the monomeric species while maintaining the electrons, resulting in deprotonated structures, notated with a dot above the atomic symbol (i.e., ^*x*^Ċ, where *x* is the atom position). The deprotonation protocol was designed to mimic the formation of plausible radicals at typical carbon-based oligomerization sites of eumelanin monomers.^35^

To assess the impact of structural conformation on the EPR signal, we investigated how the position of the hydroxyl hydrogen affects the electronic properties of the SQa monomer. The initial structure was fully optimized using the specified DFT approach. Subsequently, the hydrogen atom of the hydroxyl group was incrementally rotated in 10° steps. At each step, the resulting R–O–H dihedral angle was fixed, and the structure was reoptimized before calculating the *g*-value.

The influence of the relative orientation between adjacent monomer units on the EPR signal was also examined using DHI-based dimers (^4^/_2_ and ^4^/_7_ linkages). Each dimer was composed of one SQ and one HQ unit. The HQ unit was rotated in 10° increments, and for each configuration, the corresponding dihedral angle was fixed (the initial preoptimized R–O–H dihedral of HQ units was kept fixed throughout this process). The structures were then reoptimized prior to calculating the *g*-values.

Based on the variables identified as influencing the *g*-values, polymer chains with six units were modeled. The first unit was an SQ unit, followed by either DHI (^2^/_4_ or ^4^/_7_) or DHICA (^4^/_7_) structures. After complete optimization, the *g*-values were calculated. The other oligomers were obtained by removing one HQ unit per step, reoptimizing the system, and keeping the dihedrals fixed.

## 3 Results and Discussions

### 3.1 Polymer-Based DHI/DHICA Mixtures

The X-band CW-EPR is extensively utilized to investigate the radical species in eumelanin.^17^ As eumelanin is a mixture of DHI and DHICA units, we initially evaluated the paramagnetic environment on chemical-controlled AISSP-based DHI-rich or DHICA-rich eumelanin with different amounts of carboxylate units (DHI/DHICA ratio), Table 1.

**Table 1:** X-Band Experimental Spectral EPR Parameters Obtained from Chemical-Controlled Eumelanin Polymers

	DHI	EuM-A	EuM-B	EuM-C	DHICA
DHI/DHICA ratio (% wt)	1:0	7:3	1:1	3:7	0:1
*g*_iso_-values (±0.00001 unitless)	2.0040	2.0041	2.0041	2.0038	2.0041
Δ*H*_PP_ (±0.007 mT)	0.476	0.490	0.497	0.503	0.511
Δ*Y*_PP_ (±0.004 unitless)	1.156	1.148	1.169	1.158	1.164

At X-band frequency, DHI, DHICA, and EuM (A, B, and C) exhibited similar EPR spectra characterized by a slightly asymmetric signal and the absence of hyperfine splitting (Figure 2a). The apparent *g*_iso_-values, approximately 2.0040, were consistent across all samples, along with similar line width (Δ*H*_PP_ = 0.50 mT) and signal symmetry (Δ*Y*_PP_ = 1.16), as shown in Table 1. The obtained *g*_iso_ and Δ*H*_PP_ are compatible with previously reported natural and synthetic eumelanins.^17,21,23,36–38^ The similarities in line width might suggest that the radicals in AISSP-eumelanin are equally susceptible to hyperfine and spin–spin interactions, which could also be similar across these different samples, regardless of the number of carboxylated units.

**Figure 2:**
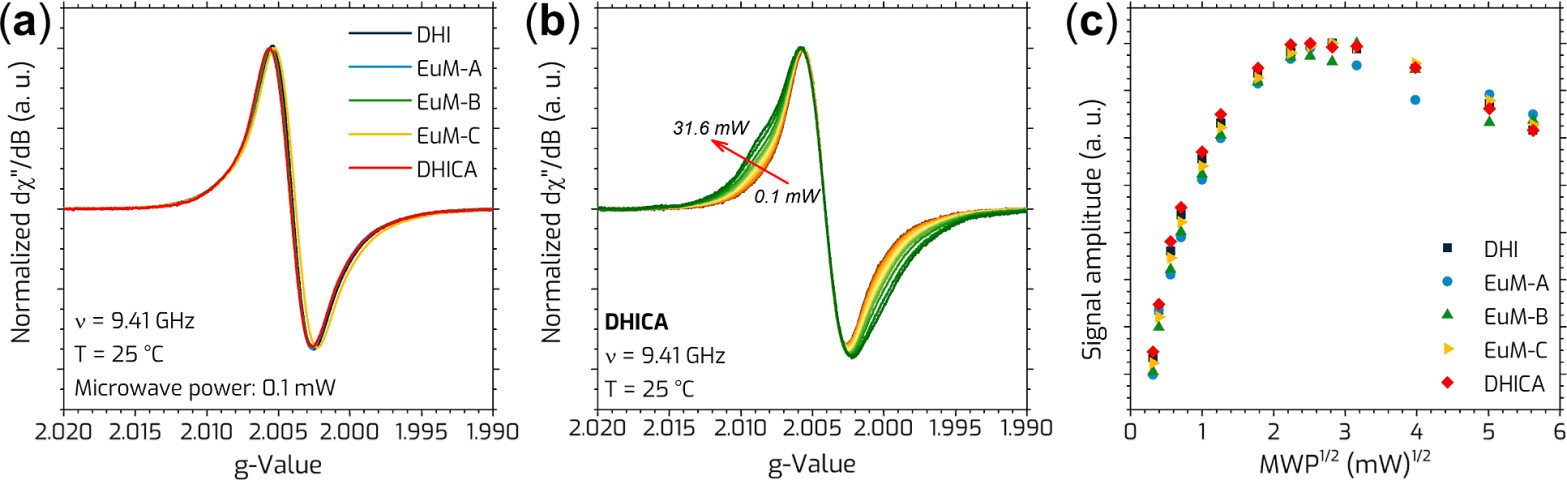
(a) CW X-band EPR spectra of different DHI and DHICA ratios measured at low microwave power (0.1 mW). The spectra are normalized to the positive peak. (b) Power saturation EPR spectra. The arrow indicates an increase in microwave power (from 0.1 to 31.6 mW). All spectra exhibit similar *g*_iso_, with the red arrow also indicating the growth of a shoulder-like feature as microwave power increases. (c) Normalized power saturation curves.

To gain deeper insights into the paramagnetic properties of our samples, power saturation experiments were conducted, ranging from negligible (0.1 mW) to relatively high (31.6 mW) saturation levels. This method enables the assessment of spin relaxation time, which can be influenced by the chemical environment surrounding the radicals.^23,28,37^ The power saturation spectra of DHICA are presented in Figure 2b. Figure S2 shows similar behavior for the other samples.

As shown in Figures 2b and S2, increasing the microwave power reveals a shoulder-like feature on the higher *g*-values side. This effect can be attributed to the presence of at least two different paramagnetic systems with similar *g*-values but slightly different spin–lattice relaxation rates.^17,37^ This hypothesis is supported by pH-dependent changes in the radical EPR response of eumelanin, which exhibits similar dependence on saturation.^19^ However, we cannot completely exclude the effects of saturation phenomena and distorted lines.^21^ This power saturation behavior aligns qualitatively with previously reported data on eumelanin.^28,37,39^

In the CW-EPR saturation experiments, *g*-values remained independent of microwave power. However, as the microwave power increased, the peak-to-peak signal showed a slight initial increase before decreasing, while the line width broadened (Figure S3). To account for variation in signal amplitude caused by the increasing line width, the saturation curve, Figure 2c, was corrected by plotting the signal intensity (i.e., area) as a function of the square root of the microwave power. This adjustment ensured a more accurate representation of the EPR signal dependence on microwave power.

A visual inspection of the saturation profile reveals that as the microwave power increases, the EPR signal intensity increases until it reaches a plateau. Beyond this point, the signal begins to decay slightly despite the continued increase in microwave power, indicating inhomogeneous broadening. While such inhomogeneous broadening was expected for DHI and EuM (A, B and C), DHICA typically demonstrates a homogeneous broadening.^23,40^ These results suggest that the inhomogeneous broadening observed in DHICA-based polymers may be linked to specific conditions during the AISSP processing, which could disrupt the usual spin distribution, possibly due to incomplete stacking or irregular bond angles formed during synthesis, affecting spin–lattice relaxation dynamics.

To further analyze the saturation effect, we applied Altenbach’s method (eq 1) for fitting. The fitting curves are presented in Figure S4, and the parameters are detailed in Table 2. Reasonable fits were obtained for all samples.

**Table 2:** Altenbach’s Fitting Parameters and Estimation of the Spin Relaxation Times

	DHI	EuM-A	EuM-B	EuM-C	DHICA
DHI/DHICA ratio (% wt)	1:0	7:3	1:1	3:7	0:1
ε (unitless)	0.67	0.66	0.69	0.67	0.67
*P*_1/2_ (mW)	3.39	3.79	3.75	3.66	3.19
*T*_2_ (ns)	13.77	13.38	13.19	13.02	12.82
*T*_2_^–1*^ (MHz)	72.60	74.76	75.83	76.78	77.99
χ^2^	0.0009	0.0011	0.0011	0.0008	0.0009

The observed ε values, close to 0.67, are compatible with the inhomogeneous broadening of the EPR line. Although no significant changes were observed in P_1/2_, it can still provide insights into the chemical variability among seemingly similar samples.^22,23^

According to Altenbach, the *P*_1/2_ may reflect the aggregation state of radical species.^22^ Thus, the variation in the *P*_1/2_ value could be attributed to differences in eumelanin-based backbone configuration. Indeed, due to the carbon positions available for polymerization (C2, C3, C4, and C7 for DHI, or C4 and C7 for DHICA, Figure 1), it is known that DHI forms globular aggregates with high π–π stacking. In contrast, DHICA has elongated rod-like aggregates assembled via weak intermolecular bundling interactions.^40^ Hence, the compact π-stacked DHI structures may be responsible for the increased *P*_1/2_ compared to DHICA. Regarding EuM (A, B, and C), the different ratios of DHI and DHICA could induce a reorganization of unpaired electrons along the polymer backbone and contribute to increased *P*_1/2_.

Moreover, the observed increase in *P*_1/2_ values in DHI-rich samples supports the hypothesis that compact π–π stacking in these structures results in a denser electronic environment,^41–43^ which influences the power required to achieve saturation. This suggests that the organization of radicals in DHI promotes stronger interactions between paramagnetic centers due to closer proximity and enhanced spin–spin coupling. Conversely, the more rod-like aggregation of DHICA reduces π-stacking efficiency,^40,44^ leading to less interaction between radicals and lower *P*_1/2_ values. These structural differences underscore how the distinct backbone configurations in DHI and DHICA impact their EPR spectral properties.

The increase in DHICA content leads to a decrease in *T*_2_ (eq 2), with the observed values aligning with the expected range for eumelanin-like materials.^38^ The strength of spin–spin interactions is influenced by the distance between paramagnetic species; as these species move farther apart, their interactions weaken due to increased separation. This phenomenon aligns with the observed reduction in *T*_2_, which suggests that the macrostructure of DHICA is smaller than that of DHI, consistent with previous reports in the literature.^44^ The variation in macrostructure size likely contributes to the differences in spin–spin interactions between these two materials.

In short, the differences in the polymer backbone and aggregation patterns between DHI and DHICA dictate the distribution and interaction of radical centers. DHI-rich polymers tend to show stronger spin–spin coupling due to compact π-stacked structures, whereas DHICA-rich polymers display more relaxed interactions in their rod-like structures.

### 3.2 Polymer vs Monomer

Figure 3 presents the X-band EPR spectra of DHI and DHICA monomers and their corresponding polymers, to examine how polymerization affects the paramagnetic properties of eumelanin-like materials. When compared to the polymer form, the apparent *g*_iso_ of the DHI monomer (Figure 3a), shifts to 2.0017 (Δ*g*_DHI_ = −0.0023), with no change in line width. Also, the signal symmetry decreases to 0.94 (Δ*Y*_PP,DHI_^′^ = −0.22) in the monomer. However, in the case of DHICA (Figure 3b), the *g*_iso_ shifts to 2.0036 (Δ*g*_DHICA_ = −0.0005), and the line width of the monomer increases to 0.85 mT (Δ*H*_PP_^′^ = +0.34). On the other hand, the signal symmetry decreases in the DHICA monomer, dropping to 0.75 (Δ*Y*_PP,DHICA_^′^ = −0.41).

**Figure 3:**
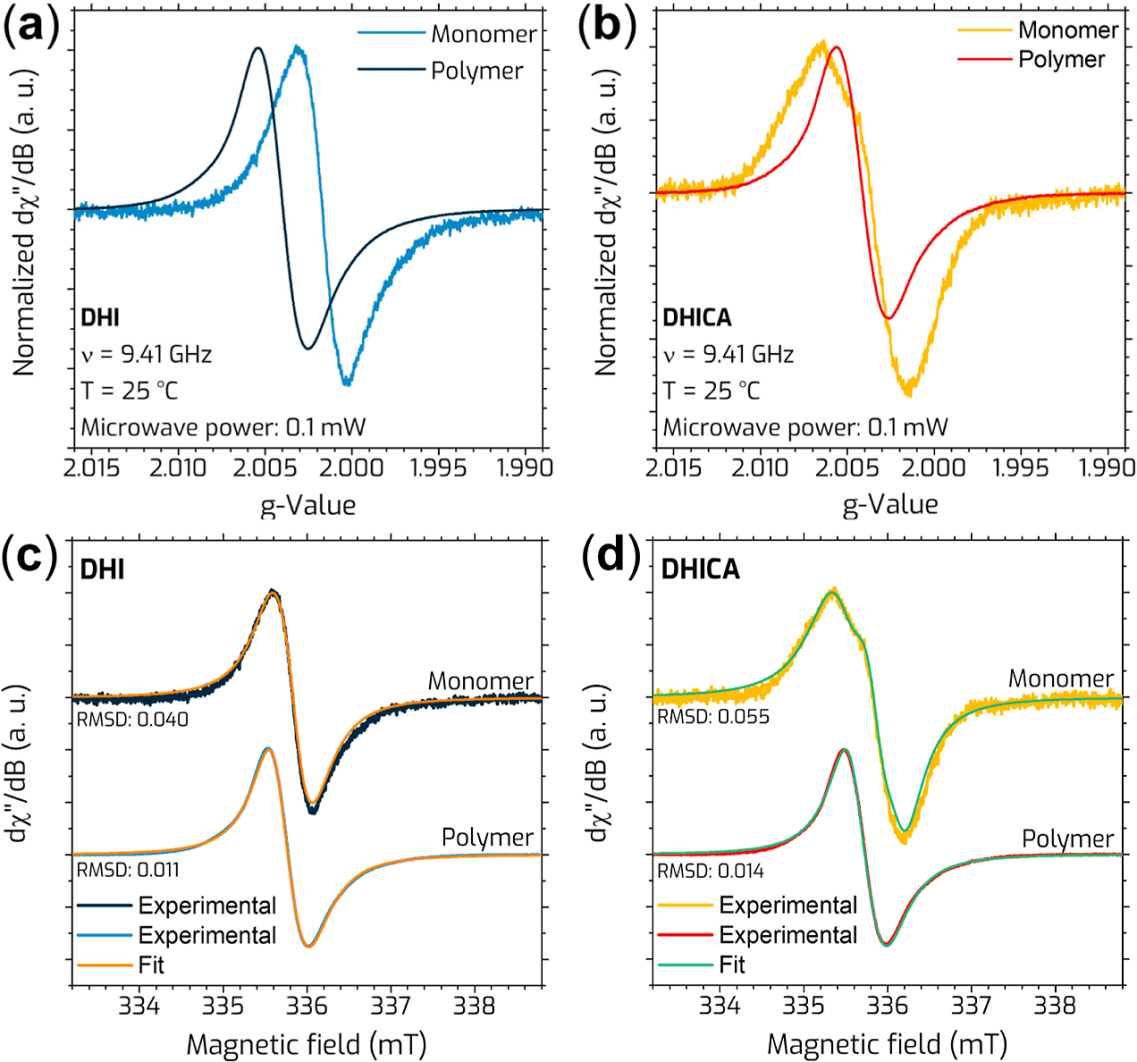
X-band EPR spectra of the monomer and polymer forms of (a) DHI and (b) DHICA, along with their corresponding fits shown in (c,d).

DHI retains its isotropic nature after polymerization, indicating that the distribution of paramagnetic centers remains uniform, and polymerization does not significantly alter the local symmetry of these centers.

In contrast, the DHICA monomer, initially characterized by axial symmetry, undergoes a transition to an isotropic form upon polymerization. This shift likely results from a structural and electronic reorganization process, potentially involving the formation of oligomeric structures in zigzag or helical conformations,^45^ which homogenize the distribution of electronic interactions in the polymer. Despite these symmetry differences, the *g*-values and line widths for both DHI and DHICA become more similar in the polymeric state, suggesting common overall magnetic properties, likely driven by the organization and packing of the polymer chains.

Spectral simulations (Figure 3c,d) were conducted to further understand these observations. The fitting parameters are summarized in Table 3. The EPR spectra of DHI monomers revealed two distinct lines, one with low *g*-values (2.0016) and higher line width (0.440 mT) and another with higher *g*-values (2.0028) and lower line width (0.337 mT). In DHI polymers, the *g* shifted toward higher values (2.0036 and 2.0045), while the line width remained similar.

**Table 3:** Optimal Fitting Parameters Obtained for DHI or DHICA Monomers and Polymers

	lines	*g*-values	Δ*H*_PP_ (mT)
DHI monomer	1	2.0016	0.440
	2	2.0028	0.337
DHI polymer	1	2.0036	0.455
	2	2.0045	0.342
DHICA monomer	1	2.0019	0.345
	2	2.0035	0.385
	3	2.0056	0.500
DHICA polymer	1	2.0037	0.455
	2	2.0047	0.342

The observed shift in *g* suggests that polymerization promotes additional magnetic interactions between DHI units, most likely due to coupling between local paramagnetic centers in the polymer network. This magnetic coupling increases the structural complexity of the polymer and enhances the density of accessible electronic states.

While EPR is highly sensitive to changes in the local electronic environment, the spectra of the polymer appear largely isotropic. This apparent isotropy does not imply the absence of local structural or electronic changes; rather, it results from the disordered nature of the polymer, where radical delocalization and the diversity of environments effectively mask anisotropic features that are otherwise evident in monomeric forms.

Three isotropic lines were considered for DHICA monomers. Although the experimental EPR signal of the DHICA monomer exhibits axial anisotropy, we use a simplified simulation with isotropic symmetry, as the axial anisotropy yields *g*-values closely matching those in the isotropic simulation (Table S1). Thus, we assume the anisotropic contributions are minor and do not significantly impact the overall interpretation.

In the case of the DHICA monomer, the spectra showed *g*-values of 2.0019, 2.0035 and 2.0056, reflecting the structural complexity and variety of electronic environments associated with different redox forms, conformations, and π-orbital arrangements within the indole system. In contrast, DHICA polymers showed *g*-values (2.0037 and 2.0047) and line widths (0.455 mT and 0.342 mT) similar to those of DHI polymers, in agreement with polymerization homogenizing the magnetic properties.

The similarity in *g*-values and line widths between DHI and DHICA polymers indicates that both types of eumelanin exhibit common magnetic characteristics in their polymeric forms. Despite possible differences in aggregation at larger scales, the local environments around unpaired electrons may be similarly disordered and magnetically coupled, resulting in comparable EPR parameters.

The *g*-values close to 2.0030 and 2.0050 can be interpreted, respectively, as the typical CCR and SFR radical species commonly observed in eumelanin systems.^18,28^ However, the signals at *g*_DHI_ = 2.0016 and *g*_DHICA_ = 2.0019 suggest EPR-active sites in monomeric units that exist in a more confined electronic environment, making them more reactive and susceptible to environmental interactions, such as oxidation.

The slightly higher *g*-value in the DHICA monomer implies that the carboxyl group may contribute to radical stabilization through inductive and hydrogen-bonding effects. The electron-withdrawing nature of the −COOH group can help delocalize spin density on the indole ring, leading to a stabilized paramagnetic species. However, this stabilization appears less effective compared to the polymeric form. Indeed, the transition from monomers to polymers in both cases results in the disappearance of the low-*g* paramagnetic species, indicating that polymerization stabilizes these paramagnetic centers. This stabilization likely arises from the delocalization of the electronic structure across the polymeric network, creating a more stable environment for radicals.

To facilitate the interpretation of the experimental results, we employed DFT-based calculations to estimate the *g*-values for different DHI and DHICA paramagnetic species, by considering: (a,b) various radical positions; (c) charged monomeric species (anionic and cationic) for HQ, IQ and QI redox forms; and (d) oligomers linked via ^4^/_2_ and ^4^/_7_ connections, containing just one SQ radical unit at the beginning of the chain (Figure 4). Specific *g*-values for all these species are provided in Tables S2–S4.

**Figure 4:**
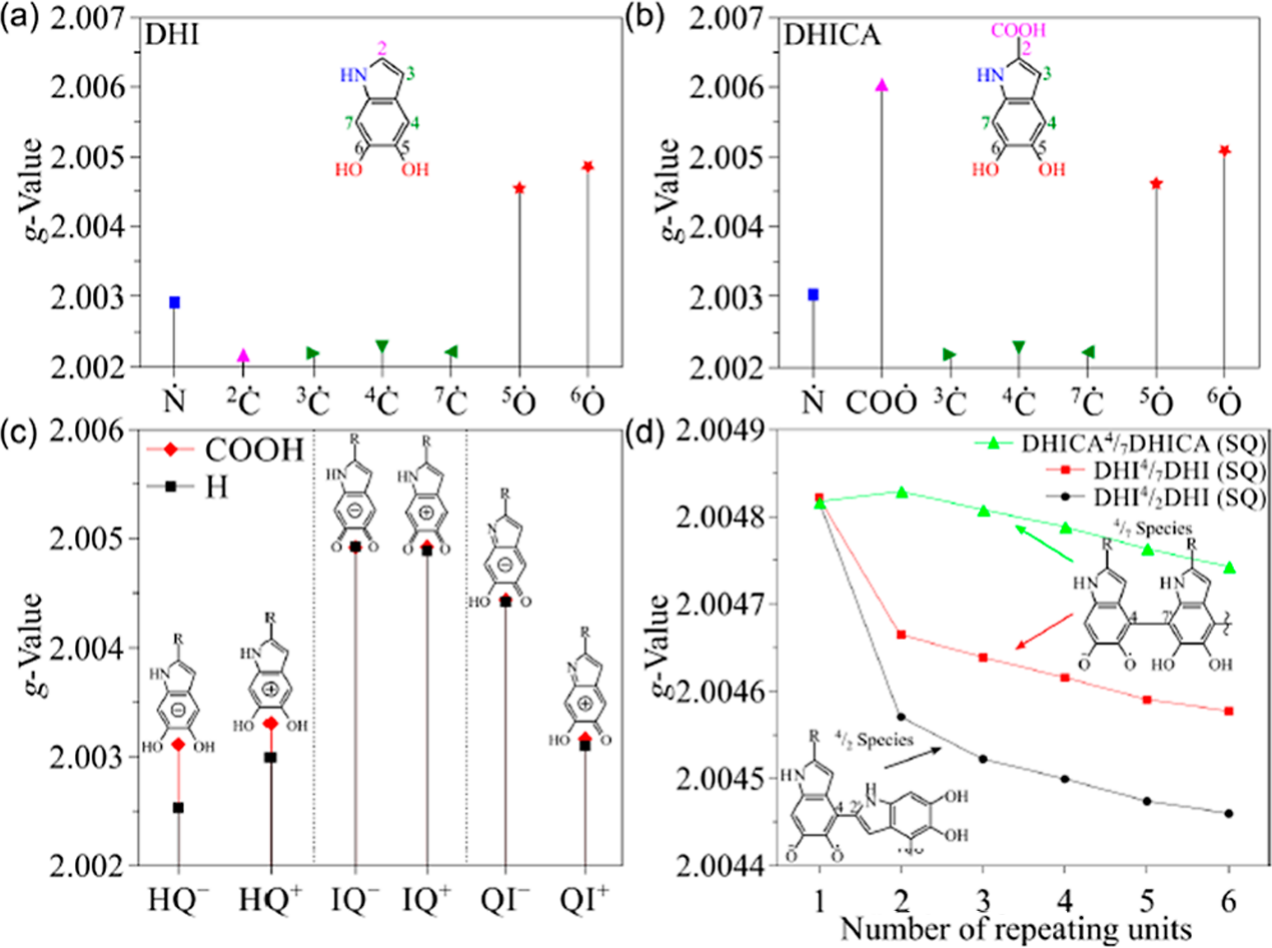
*g*-Values of various species derived from DHI and DHICA. (a) Deprotonated DHI species. (b) Deprotonated DHICA species. (c) Charged species resulting from oxidation and reduction processes. (d) Effect of increasing the number of repeating units on *g*-values, using SQ units at the initial position.

Low *g*-values are obtained for CCR, with similar results for DHI and DHICA. Note that the experimental low *g*-values (2.0016 and 2.0019) are smaller than those of the evaluated structures, being closest to C2 (for DHI), C3, C4, and C7 centered radicals, Figure 4a,b. The detection of such low *g*-value EPR-active sites in monomeric units (undetectable in oligomers) suggests that these species exist in a confined and possibly reactive electronic environment that likely enhances their chemical reactivity. This supports the hypothesis that such paramagnetic centers are consumed during polymerization, reinforcing their possible association with the C-centered radicals. However, the exact lifetime and reactivity of these centers remain undetermined in this study.

Species with intermediate *g*-values (2.0028 and 2.0035) can be linked to nitrogen-centered and/or cationic (e.g., HQ^+^ and QI^+^) species. These paramagnetic centers are compatible with CCR species as reported elsewhere.^18,29,37^ In particular, from Figure 4c, the existence of systems with relative high (IQ^+^, IQ^–^, and QI^–^) and low (HQ^+^, HQ^–^, and QI^+^) *g*-values is noted. Anionic species such as HQ^–^ and IQ^–^ (DHI) can be considered unlikely structures, given the positive energy of their semioccupied molecular orbitals (Figure S6).

The high *g*-value 2.0056, observed only for DHICA monomers, aligns well with oxygen-centered SFR (SQ, SQa) and DHICA-COO^–^ (Figure 4b–d), and could indicate the stabilization of semiquinones in such monomers or the plausibility of COO^–^-centered radicals in these systems. It is worth mentioning that SQa *g*-values (oxygen-centered radicals) can present a significant variation (from ∼2.0045 up to ∼2.0054) depending on the hydrogen dihedral angle, as shown in Figure S7.

After polymerization, only two experimental lines are identified for DHI and DHICA-based systems. The low *g*-values (2.0036 and 2.0037) can be linked to CCR, which can be associated with HQ^+^, QI^+^ and nitrogen-centered species, while higher values (2.0045 and 2.0047) can be connected to oxygen-centered SFR.^18^ The observation of SFR in polymers (not present in DHI monomers) suggests that the extended conjugation in the polymeric network may contribute to the stabilization of O-centered radicals. However, alternative interpretations cannot be ruled out at the moment.

The *g-*value of DHI SQ-based oligomers exhibits a gradual and continuous decrease with the number of oligomeric units, while DHICA-based ones follow a more complex pattern, also presenting an asymptotic behavior for long chains. The DHI species exhibit lower *g*-values than the DHICA species, which is compatible with the experimental results. The saturated values, estimated using a double exponential decay model, are 2.00438 (DHI^4^/_2_DHI), 2.00450 (DHI^4^/_7_DHI), and 2.00467 (DHICA^4^/_7_DHICA), all of which are in good agreement with the experimental data.

Interestingly, the changes induced by oligomer growth fall within the same range as those caused by variations in the relative positions of adjacent units (from 2.0043 to 2.0048, Figure S8). Such changes can lead to unresolved spectra (Δ*g* ∼ 0.0005). More significant changes due to polymer conformation are supposed to occur for ^4^/_7_ connected oligomers, once low energy conformers present the highest d*g*/dθ relationships (with θ representing the dihedral between adjacent units, Figure S8). This is in line with the higher Δ*H*_PP_ values noticed for DHICA-rich samples (Table 1).

The computational findings align with the experimental EPR data, suggesting that polymerization homogenizes the electronic environment of eumelanins and that the polymer structure can influence the distribution of unpaired electrons. The spectral changes observed upon polymerization, particularly in DHICA, suggest that electronic and structural reorganization enhances the uniformity of electronic interactions within the polymer.

In particular, the polymerization process eliminates (or, at least, destabilizes) low-*g* systems, stabilizing other radical centers. This stabilization, along with the spectral homogenization, reinforces the role of polymerization in shaping the electronic and magnetic properties of eumelanin materials.

The variation in *g*-value observed in Figure 3 could also potentially arise from spin–orbit coupling,^46^ influenced by the polarization of functional groups such as amine, carboxyl, and hydroxyl (Figure 1). However, Figure 4 suggests that if the paramagnetic species were closely associated with these groups, the *g* would align with SFR signals, which are significantly higher than the simulated values. This supports our interpretation that these shifts stem from local electronic interactions and structural reorganizations.

To better contextualize these observations, Table 4 summarizes the main types of paramagnetic species identified in DHI and DHICA, along with their corresponding g-value derived from experimental EPR data analysis and DFT calculations.

**Table 4:** Overview of Radical Types and Corresponding *g*-Values for DHI and DHICA Monomers and Polymers

radical type	associated structure	*g*-value (experimental)	*g*-value (theoretical)^a^	notes
DHI				
CCR	monomer	2.0016	2.0022–2.0023	confined local electronic environment; disappears after polymerization
N-centered/cationic radicals	monomer	2.0028	2.0029–2.0031	associated with Ṅ, HQ^+^ and QI^+^ species
CCR	polymer	2.0036	2.0035–2.0037	stabilized and homogenized after polymerization
SFR	polymer	2.0045	2.0045–2.0054	stabilized by extended conjugation
DHICA				
CCR	monomer	2.0019	2.0019–2.0020	confined local electronic environment; radical stabilized by the COOH; disappears after polymerization
N-centered/cationic radicals	monomer	2.0035	2.0030–2.0033	associated with Ṅ, HQ^+^ and/or QI^+^ species
SFR	monomer	2.0056	2.0046–2.0060	associated with SQ and COO^–^ radical
CCR	polymer	2.0037	2.0036–2.0038	polymerization homogenizes interactions
SFR	polymer	2.0047	2.0045–2.0054	stabilized through polymerization

a Based on values found here and Batagin-Neto et al. (2015).^18^

Overall, polymerization acts to stabilize paramagnetic centers and homogenizes electronic interactions, indicating that the connectivity and structural organization of the polymer play a decisive role in defining the magnetic and electronic properties of eumelanin.

These findings offer insights into the electronic behavior of eumelanin. The stabilization of EPR-active species and reduced differences between DHI and DHICA in polymeric forms suggest that connectivity and structural organization govern electronic delocalization. While these results do not directly confirm charge transport mechanisms, the spectral homogenization observed after polymerization supports the hypothesis that chain organization could influence charge mobility. This speculative correlation aligns with experimental observations linking eumelanin’s conductivity to hydration and supramolecular structure.^1,47^ However, this interpretation remains speculative and should be further investigated through dedicated hydration-dependent charge-transport studies.

In the starting material (likely largely monomeric form but other species cannot be excluded), low-*g* signals (2.0016 for DHI and 2.0019 for DHICA) are observed by CW-EPR but vanish once polymerization takes place. This would suggest the presence of localized paramagnetic centers in the monomeric species, which are either destabilized or consumed during polymer growth. While CW-EPR provides only time-averaged information and cannot capture ultrashort-lived intermediates,^48,49^ the fact that these signals are detected under steady-state conditions indicates that these species are stable enough to be observed. A possible support to monomeric paramagnetic centers comes from DFT calculations, which assign comparable *g*-values to CCR in monomeric units. By contrast, the polymeric forms yield more uniform spectra, reflecting the stabilization and delocalization of these species across the extended network.

Time-resolved EPR approaches, not employed in this work, could help monitor their evolution in real time, offering more profound insight into the electronic changes that accompany polymer growth. Complementary temperature-dependent and pulsed EPR measurements (e.g., *T*_1_/*T*_2_ echo and electron–nuclear double resonance) could further elucidate the spin relaxation and coherence dynamics. In situ UV–vis or vibrational spectroscopies during AISSP may also assist in tracking the evolution of functional groups. While these techniques are beyond the scope of the present study, they represent promising tools for future investigations into the kinetics and mechanisms of eumelanin formation.

## 4 Conclusion

This study provides a comprehensive understanding of how the polymerization and molecular composition of DHI and DHICA monomers significantly influence the paramagnetic properties of eumelanin. Through X-band CW-EPR analysis, the research reveals that varying the DHI/DHICA ratio does not substantially alter the free radical composition, spin dynamics, and relaxation times within the material. We also showed a strong dependence of the EPR signal on the deprotonation site and chain length. These findings highlight an intricate balance between local electronic structure and long-range organization, ultimately defining the eumelanin’s electronic and paramagnetic properties. The study underscores that the transition from monomeric to polymeric states introduces substantial changes in spin interactions and the overall electronic environment, offering valuable insights into how these values impact its physicochemical behavior.

Beyond their fundamental implications, these findings also shed light on the potential applications of eumelanin in biomedicine and bioelectronics. The stabilization of radicals and the electronic uniformity that emerges after polymerization suggest improved charge delocalization, a key aspect for conductivity and charge transport in eumelanin-based devices. The polymer network’s ability to stabilize unpaired spins may also help explain its well-known photoprotective and antioxidant behavior. Altogether, these insights demonstrate how a deeper understanding of eumelanin’s molecular and electronic organization can guide the development of sustainable bioelectronic materials and inspire novel approaches to functional biomimetic systems.

## Supplementary Material



## Notes

Additional EPR analyses; signal simulations; electronic structure calculations data; additional electronic structure calculations (PDF)
