# Supplementary material for: In-Depth Analysis of the Paramagnetic Properties in DHI/DHICA-Controlled Eumelanin
Source: ACS Omega. 2025 Nov 7;10(45):54919–28. doi: 10.1021/acsomega.5c08896 (PMC12631427; doi:10.1021/acsomega.5c08896)
Supplement: Supplementary file 1 [file ao5c08896_si_001.pdf]

Supporting Information

In-Depth Analysis of the Paramagnetic Properties in DHI/DHICA-controlled Eumelanin

João V. Paulin,[a,*] João P. Cachaneski-Lopes,[b] Emanuele Carrella,[c] Alessandro Pezzella,[d,e,f] Augusto Batagin-Neto,[b,g] Carlos F. O. Graeff[a,b,*]

[a] São Paulo State University (UNESP), School of Sciences, Department of Physics and Meteorology, Bauru/SP, Brazil.

[b] São Paulo State University (UNESP), Graduate Program in Materials Science and Technology (POSMAT), Bauru/SP, Brazil.

[c] Department of Chemical Sciences, University of Naples Federico II, via Cinthia 4, 80126 Naples, Italy.

[d] Department of Physics Ettore Pancini, University of Naples Federico II, Via Vicinale Cupa Cintia, 21, Naples, 80126 Italy

[e] Institute for Polymers Composites and Biomaterials (IPCB) CNR, Via Campi Flegrei 34, IT-80078 Pozzuoli (Na), Italy and Bioelectronics Task Force at University of Naples Federico II, Naples, Italy

[f] National Interuniversity Consortium of Materials Science and Technology (INSTM), Piazza S. Marco, 4, Florence, Naples 50121, Italy

[g] São Paulo State University (UNESP), Institute of Science and Engineering, Itapeva/SP, Brazil.

S1. Additional EPR data

Figure S1 shows the results coming from CW EPR spectra simulation via the EasySpin computational package. The Pepper routine was employed, considering Gaussian, Lorentzian, and Voigtian line shapes. Distinct anisotropies (isotropic or axial symmetry), and the number of species were also considered.

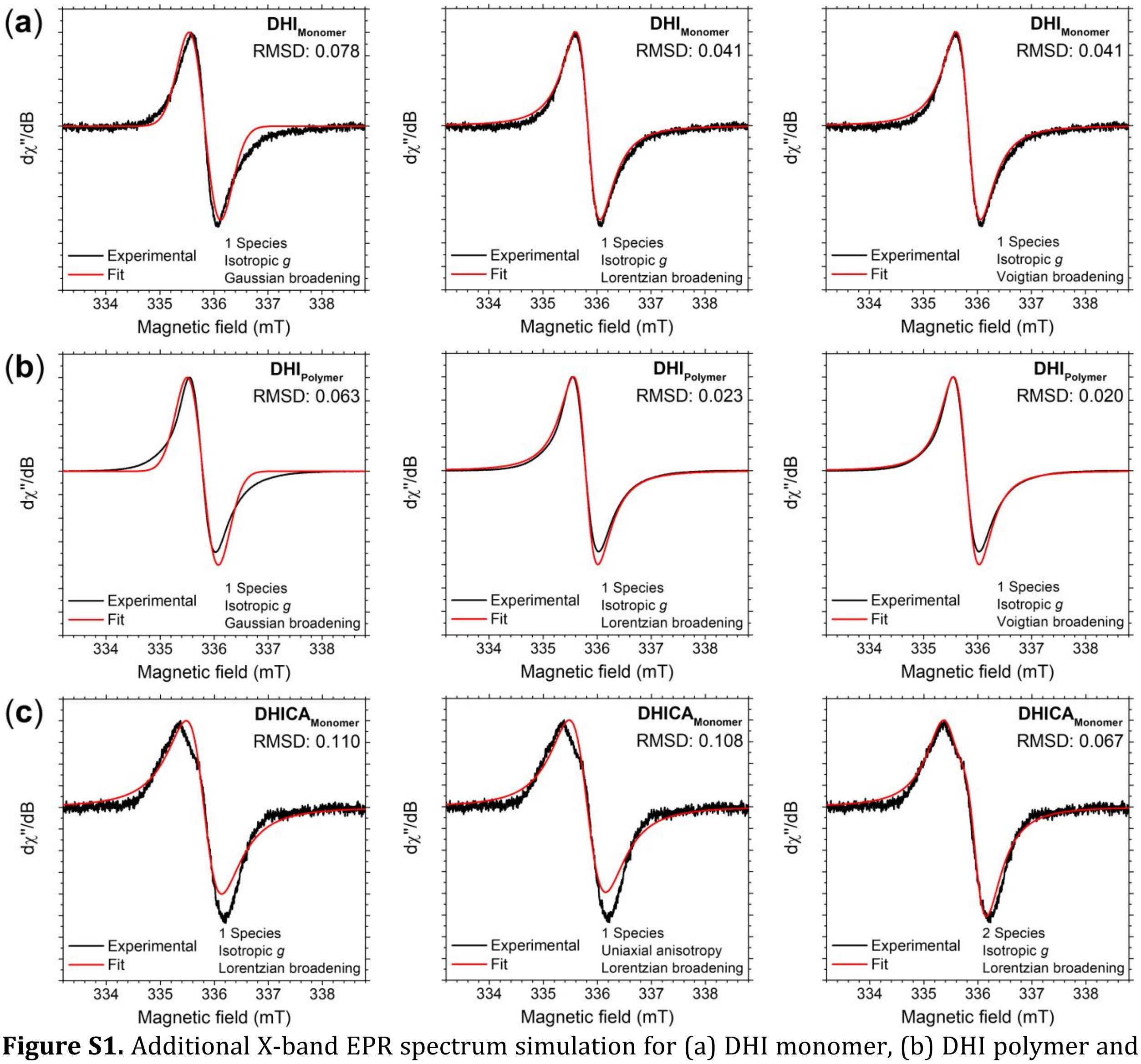

Figure S1. Additional X-band EPR spectrum simulation for (a) DHI monomer, (b) DHI polymer and (c) DHICA monomer.

Figure S2 shows the CW X-band EPR spectra of different eumelanin samples measured at distinct microwave powers, from 0.1 to 31.6 mW (arrows indicate increasing microwave power). Figures S3 and S4 show power saturation parameters.

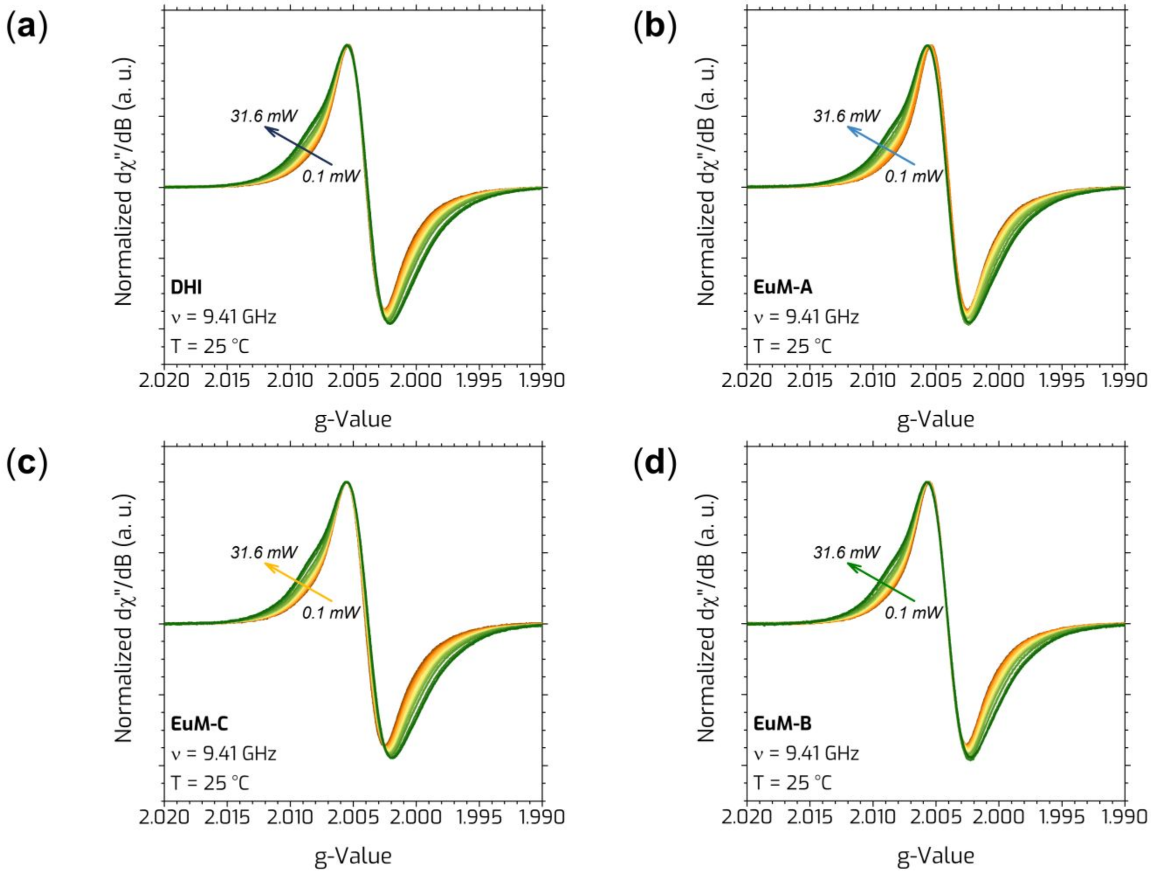

Figure S2. Power saturation EPR spectra of (a) DHI, (b) EuM-A, (c) EuM-B, (d) EuM-C.

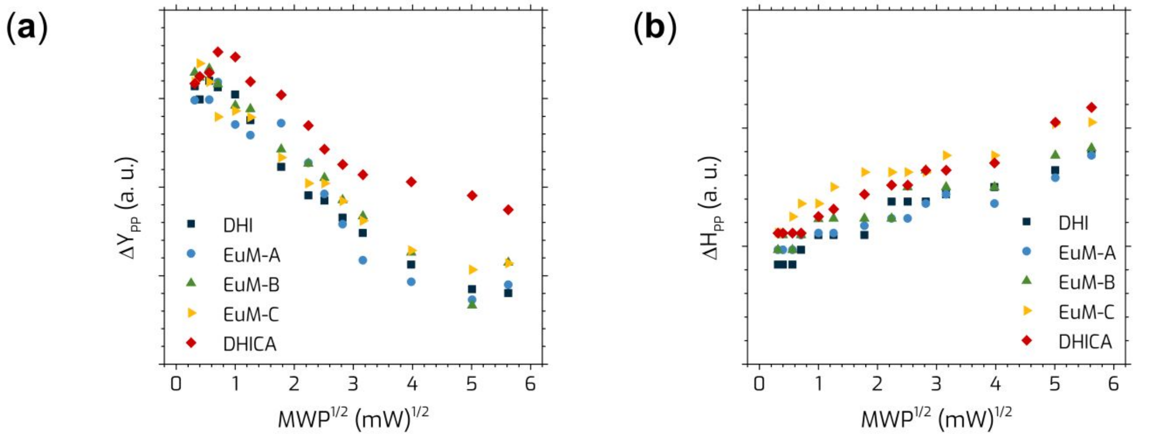

Figure S3. (a) Peak-to-peak intensity and (b) linewidth as a function of the square root of the microwave power.

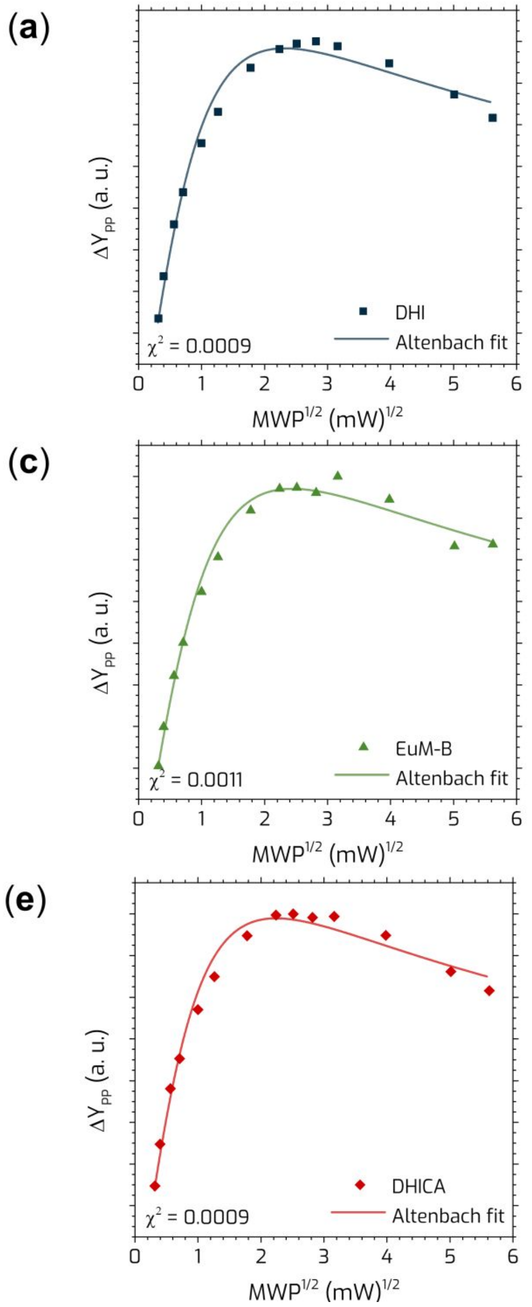

Figure S4. Power saturation curve numerical fitting to Eq. 01 (main text) of (a) DHI, (b) EuM-A, (c) EuM-B, (d) EuM-C, and (e) DHICA.

Table S1. Fitting parameters obtained for DHICA monomers with axial symmetry.

	Lines	g-Values	ΔH_{PP} (mT)
DHICA Monomer	1	2.0035/2.0011	0.318
	2	2.0058/2.0033	0.494

S2. Parameters estimated via DFT calculations: Theoretical data

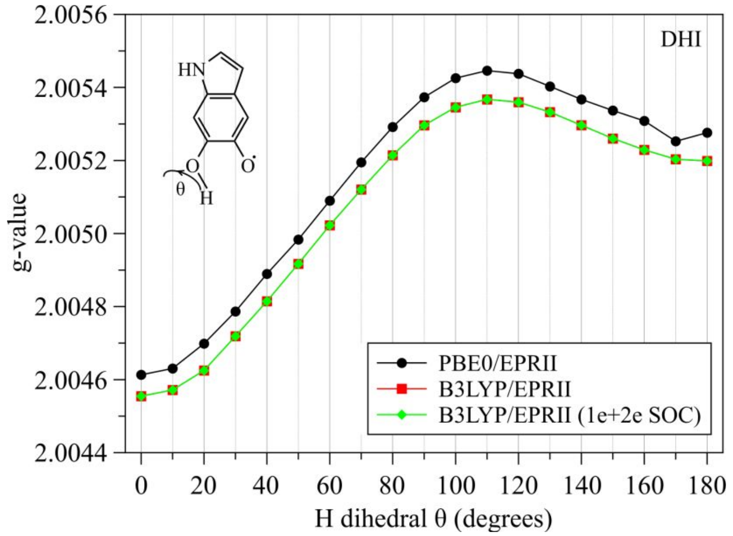

Figure S5. Calculated g-values for the DHI radical as a function of the hydroxyl hydrogen dihedral angle (θ). Results are shown for different levels of theory: PBE0/EPR-II (black), B3LYP/EPR-II (red), and B3LYP/EPR-II including one- and two-electron spin–orbit coupling (green).

Tables S2-S4 present the g-values for different DHI and DHICA radical species, considering distinct radical positions (S2), charged HQ, IQ and QI species (S3) and SQ-based oligomeric systems (S4).

Table S2. Numerical g-values for deprotonated DHI and DHICA monomers.

Structure	DHI
Ṅ	2.0029119
$^{2}\dot{\mathrm{C}}$	2.0021753
$^{3}\dot{\mathrm{C}}$	2.0021963
$^{4}\dot{\mathrm{C}}$	2.0022852
$^{7}\dot{\mathrm{C}}$	2.0022199
$^{5}\dot{\mathrm{O}}$	2.0045534
$^{6}\dot{\mathrm{O}}$	2.0048483

Structure	DHICA
Ṅ	2.0030380
COȮ	2.0060409
$^{3}\dot{\mathrm{C}}$	2.0021855
$^{4}\dot{\mathrm{C}}$	2.0022825
$^{7}\dot{\mathrm{C}}$	2.0022251
$^{5}\dot{\mathrm{O}}$	2.0046244
$^{6}\dot{\mathrm{O}}$	2.0050806

Table S3. Numerical g-values of oxidized and reduced monomeric species of DHI and DHICA.

Structure	DHI	DHICA
SQa	2.00520	2.00523
SQb	2.00485	2.00508
IQ^-	2.00492	2.00492
IQ^+	2.00489	2.00492
QI^-	2.00442	2.00444
QI^+	2.00310	2.00316
HQ^-	2.00253	2.00311
HQ^+	2.00299	2.00331

Table S3. Numerical g-values for oligomers linked via C4-C2 ($^4/_2$) or C4-C7 ($^4/_7$) sites.

Number of repeating units	DHI $^4/_2$ DHI (SQ)	DHI $^4/_7$ DHI (SQ)	DHICA $^4/_7$ DHI (SQ)
1	2.00482	2.00482	2.00482
2	2.00457	2.00467	2.00483
3	2.00452	2.00464	2.00481
4	2.00450	2.00461	2.00479
5	2.00447	2.00459	2.00476
6	2.00446	2.00458	2.00474

Figure S6 presents the frontier energy levels (HOMO, LUMO or SOMO) of neutral and charged monomeric species (anionic and cationic) of DHI and DHICA for the distinct forms: HQ, IQ and QI. As one can see, DHI-HQ^-, DHI-IQ^- and DHICA-HQ^- present very high HOMO energies (HOMO > 0 eV), making them unstable species.

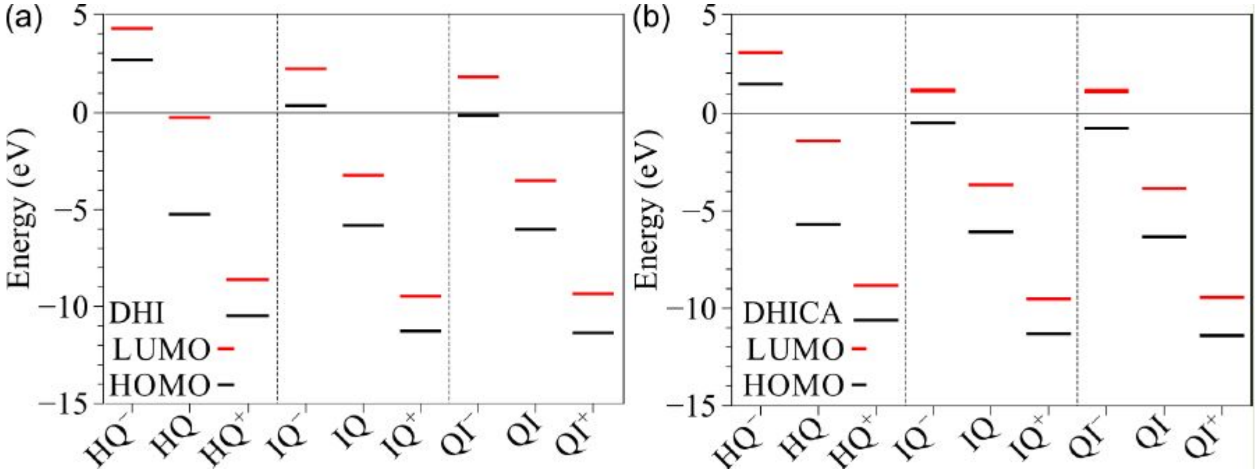

Figure S6. The frontier molecular orbital of anionic and cationic monomers of (a) DHI and (d) DHICA.

Additional studies were conducted in monomeric and dimeric structures to investigate how structural conformation affects the EPR signal. We first examined the influence of the hydroxyl hydrogen position in the SQa (protonated semiquinone) monomer. The structure was fully optimized using the DFT approach (described in the *Main Text*), and the hydrogen atom was rotated in 10° increments. At each step, the optimization and EPR spectrum were calculated. The results are displayed in Figure S7.

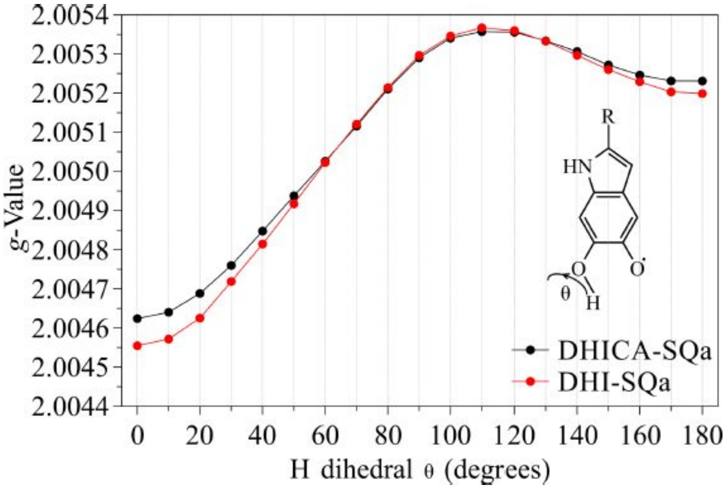

Figure S7. The influence of hydrogen (from OH) on the SQa rotation on the monomer g-value.

We also evaluated the effect of the relative position between two DHI monomer units on the EPR signal (Figure S8). For this, dimers connected at positions C4-C2 and C4-C7 were studied. Each

dimer consisted of one SQ unit and one DHI one. The DHI unit was rotated in 10° increments, and the optimization and the EPR spectrum were computed at each step.

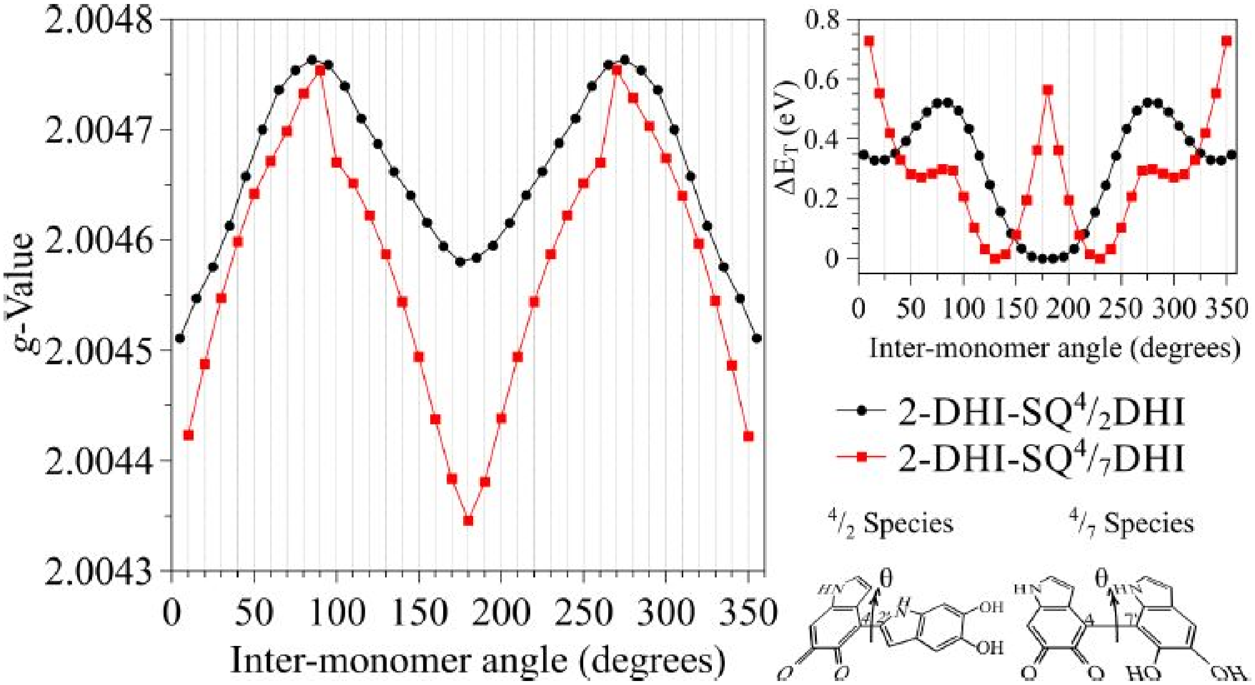

Figure S8. Influence of the relative position of the monomer in dimers on EPR.

The g-values of species $^4/_7$ are more sensitive to the dimer's structural conformation (Figure S7), with g varying around 0.0008, while species $^4/_2$ exhibits a variation of 0.0007. The inset on the right of Figure S8 shows how the total energy of the molecule varies in relation to its lowest energy during the rotation process. The lowest energy conformation of $^4/_2$ species is observed for around 170-180°, with two local minima around 10-15° and 340-350°. $^4/_7$ species present local minimum at 50-70° and 290-310°, and global minimum at ~130° and ~230°.

The g-value derivative in relation to inter-monomer angle (θ) is minimum at the global minimum of $^4/_2$, indicating that small changes are expected due to thermal conformational fluctuations. On the other hand, $^4/_7$ species present high dg/dθ around their global minimum, suggesting a more effective effect of conformational fluctuations around the equilibrium.
